# Fruit and vegetable consumption, leisure‐time physical activity, and sedentary behavior among children and adolescent students in Uganda

**DOI:** 10.1002/fsn3.883

**Published:** 2019-01-24

**Authors:** Catherine T. Ndagire, John H. Muyonga, Dorothy Nakimbugwe

**Affiliations:** ^1^ School of Food Technology, Nutrition and Bio‐engineering Makerere University Kampala Uganda

**Keywords:** adolescents, children, fruit and vegetable consumption, physical activity, sedentary behavior

## Abstract

**Introduction:**

Fruit and vegetable (FV) intake and participation in leisure‐time physical activity (PA) and in sedentary behavior in accordance with the recommendations are met in a minority of populations including children and adolescents.

**Objective:**

To describe the frequency of FV intake, leisure‐time PA, and sedentary behavior, assess compliance with international recommendations, and how selected factors are associated with these recommendations.

**Methods:**

The FV consumption and PA questionnaire items previously assessed for reliability in a Ugandan setting were used to gather information about FV consumption, PA, and sedentary behavior among 621 children and adolescents attending schools in Kampala Uganda. The results of FV consumption and PA were categorized as adequate or inadequate while sedentary behavior was categorized as adequate or excess. The relationship between FV consumption, PA, and sedentary behavior with selected independent variables was verified by chi‐square test statistic.

**Results:**

Half of the participants (50%) reported inadequate fruit consumption, and 76% of them reported inadequate vegetable consumption. More children (68%) than young adolescents (52%) and older adolescents (44%) met their daily recommendation for fruit consumption. A similar trend was observed for vegetable consumption. Adequacy of FV intake was significantly associated with participant's age, level of education, school ownership, and type of school. Less of 8‐ to 17‐year‐olds (14%) than 18 years and above olds (41%) met their weekly recommendation for leisure‐time PA. Majority of participants (54%) participated in acceptable levels of sedentary behaviors. Leisure‐time PA was significantly associated with age category and school ownership while sedentary behavior was significantly associated with participant's age and education level.

**Conclusions:**

The intake of FV and participation in leisure‐time PA was not adequate in the study population. A significant proportion of participants engaged in excess sedentary behavior. Developing and implementing strategies to increase FV intake and PA among children and adolescents in schools should be a national priority.

## 1 INTRODUCTION

School‐age children and adolescents have an increased need for nutrients because their growth and development are dynamic (Ochola & Masibo, 2014). Good nutrition during adolescence is critical to cover any deficits suffered during childhood. This should also include nutrients required to meet the demands of physical and cognitive growth and development, provide adequate stores of energy for illnesses and pregnancy, and prevent adult onset of nutrition‐related diseases (Erkan, 2011). Many children's and adolescents’ diets are inadequate with respect to fruit and vegetable (FV) intake. Thus, the diets of children and adolescents are most likely to be low in micronutrients, fiber, and are consequently associated with a high waist circumference and therefore overweight and obesity (Francis et al., 2009). Moreover, excess weight in children is associated with reduced quality of life and increased risk of negative emotional states, undesirable stereotyping, bullying, and social isolation (Roura, Milà‐Villarroel, Pareja, & Caballero, 2016). The World Health Organization (WHO) estimates that 2.7 million lives which are lost due to cardiovascular diseases could be prevented if FV intake is adequate (Agudo, 2005). At the same time, available data indicate an emerging trend of consumption of high‐energy snacks and beverages and low FV intake, particularly in urban areas (Ochola & Masibo, 2014). The Westernized type of diet has become increasingly common among adolescents in developing countries and may be implicated in the emerging regional obesity epidemic (Francis et al., 2009).

Fruits and vegetables have beneficial effects on health since they provide vitamins, minerals, fiber, and other nutrients such as photo‐chemicals with protective effects, stimulating the immune system and other physiological systems (Ramírez‐Silva, Rivera, Ponce, & Hernández‐Avila, 2009). Adequate FV consumption is also associated primarily or secondarily with less incidence of obesity, cardiovascular disease, diabetes mellitus type II, and several types of cancer (Ramírez‐Silva et al., 2009). In spite of the importance of an adequate FV intake during childhood and adolescence and the consequences of their inadequacy, children and adolescents in most Western countries, Asian countries, Costa Rica, and African countries eat far less than the recommended amount of fruits and vegetables (Peltzer & Pengpid, 2012). The United States Department of Health and Human Services recommends at least two daily servings of fruits and three daily servings of vegetables (Peltzer & Pengpid, 2012).

Adequate promotion of PA is an essential prerequisite for motor development and enhances psychological and social health outcomes in children. It is also associated with numerous health benefits against physical (e.g., obesity) and psychological effects (e.g., depression) and even with health conditions such as metabolic syndrome during adulthood (Götte, Seidel, Kesting, Rosenbaum, & Boos, 2017). PA levels have decreased while sedentary behaviors such as television viewing and playing video/computer games have increased, yet low‐intensity physical activity is associated with obesity in adolescents (Li et al., 2007). World Health Organization (WHO) recommends that children and youth aged 5–17 years should accumulate at least 60 min of moderate‐ to vigorous‐intensity PA daily (WHO, 2017b) while adults aged 18–64 should do at least 150 min of moderate‐intensity aerobic PA throughout the week (WHO, 2017a). Increasing PA and reducing sedentary behavior play important roles in health promotion and prevention of lifestyle‐related diseases in children and adolescents (Graf et al., 2014). However, PA among children and adolescents is believed to be insufficient, and low levels of PA seem to persist into adulthood. This makes physical inactivity among young people a risk factor for cardiovascular disease, cancer, and osteoporosis in later life (van Sluijs, McMinn, & Griffin, 2007).

Sedentary lifestyle patterns in children and adolescents, that is, playing digital games, using computers and especially watching television, have been associated with obesity (Rey‐López, Vicente‐Rodríguez, Biosca, & Moreno, 2008). In children and adolescents, independent of PA levels, sedentary behaviors are associated with increased risk of cardio‐metabolic disease, all‐cause mortality, and a variety of physiological and psychological problems (Sisson et al., 2009; Tremblay et al., 2011). Therefore, to maximize health benefits, approaches to resolve the inactivity crisis should attempt to both increase deliberate PA and decrease sedentary behaviors. However, to date, public health efforts have focused primarily on PA and have paid little attention to the mounting evidence to support sedentary behavior as a distinct behavior related to poor health. The American Academy of Pediatrics (AAP) recommends no more than 2 hr a day of screen‐based activities (e.g., TV viewing, computer games, and console games) due to the relationship between screen time and health risk (Martínez Gómez et al., 2012; Sisson et al., 2009).

Given that food intake patterns established during childhood may track into adulthood and the potential associations of FV intake with other eating and health behaviors (e.g., physical activity and sedentary behavior) (Chong, Lee, Ng, Khouw, & Poh, 2017) and the importance of increasing PA and reducing sedentary behavior in health promotion and prevention of lifestyle‐related diseases in children and adolescents (Graf et al., 2014), increasing the intake of FV, PA, and reducing sedentary behavior among children and adolescents should therefore be public health priorities. In 2003, 19.8% of Ugandan school children and adolescents ate fruits and vegetables five or more times per day during the past 30 days, only 15.3% were physically active all the previous 7 days for at least 60 min continuously, 27.5% spent 3 hr or more per day doing sitting activities such as watching TV/video or playing computer games (Twa‐Twa & Oketcho, 2005). There is limited up‐to‐date published data available on FV consumption, PA, and sedentary behavior among children and adolescents, collected using reliable tools in Uganda. In addition, little is known about the extent to which the FV consumption, PA, and sedentary behavior comply with international recommendations and their association with selected factors. A better understanding of these associations is particularly important for providing insights toward the development of more effective strategies and approaches to target the double burden of malnutrition reported in many developing countries, including Uganda (Abdullah, 2015). Thus, the purpose of the current study was to determine the frequency of FV consumption, engagement in leisure‐time PA, and sedentary behavior among children and adolescents aged 8–19 years attending schools in Uganda and subsequently assess compliance with international recommendations.

## 2 METHODS AND MATERIALS

### 2.1 Subjects

Students aged 8–19 years, attending primary and secondary schools in Kampala district, Uganda, were recruited as participants for this study. A representative sample of participants was obtained through multiple‐stage sampling in the order of Kampala district division, school, and class. Twelve representative schools were eventually selected to reflect the population distribution in Kampala city schools.

### 2.2 Sample size calculation

Sample size was calculated using Daniel's equation as illustrated below:

$$n=\frac{t^{2}\times p\left(1-p\right)}{m^{2}}$$

Where: *N* is the required sample size, *t* is the confidence level at 95% (standard value of 1.96), *p* is the estimated prevalence of malnutrition in the project area.

For this study, *p* = 0.802; the proportion of school children aged 13–15 years who reported inadequate FV consumption during the Global school‐based health survey in Uganda (Twa‐Twa & Oketcho, 2005)

*m* = margin of error at 5% (standard value of 0.05).

### 2.3 Base sample size

*n* = 244.0123, approximately 245

### 2.4 Design effect (*D*)

Since the study used cluster sampling methodology, *D* = 2 was assumed as this is the recommendation for nutrition surveys using cluster sampling methodology (IFAD, 1995).

= *n* × 2

= 245 × 2

= 490

### 2.5 Contingency

The sample was increased by 25% to account for contingencies such as nonresponse

*n* = 490+ (0.25 × 490)

*n* = 612.5

### 2.6 Assessment of FV consumption, PA, and sedentary behavior

The FV consumption and PA questionnaire items were adopted from the Uganda Global Schools’ Health Survey questionnaire (Twa‐Twa & Oketcho, 2005). These items had been previously assessed for reliability using test–retest reliability method in a Ugandan setting were used to gather information about FV consumption, leisure‐time PA, and sedentary behaviors among 621 children and adolescents attending schools in Kampala, Uganda. Slight modifications were made on the PA items. Demographic data including respondents’ age, sex, level of education, class attended in school, school ownership, and type of school attended were also collected. The FV consumption tool had two items on frequency of FV consumption in the past 30 days: number of times per day that participants usually ate fruits such as ripe bananas, mangoes, oranges, guavas, avocado, or pawpaw in the past 30 days and number of times per day that participants usually ate vegetables, such as *dodo, gobe, ntula, sekuma wiki, buga, or nakati* in the past 30 days. The PA tool had seven items that captured: number of days on which respondents were physically active for at least 60 min per day in the past 7 days, number of days on which respondents were physically active for at least 60 min per day during a typical or usual week, time spent in sedentary behaviors, number of days the respondent walked or rode a bicycle to and from school during the past 7 days, time respondents usually spent to and from school each day during the past 7 days, number of days respondents did exercises to strengthen or tone their muscles during the past 7 days, and how respondents felt after PA. Participants aged above 17 years were asked for number of minutes in a week they engaged in moderate‐ to vigorous‐intensity instead of number of days on which they were physically active for at least 60 min per day in the past 7 days, to suit the WHO PA recommendations. The rest of the data were categorized based on WHO's standard age group categorization: 5–9, 10–14, and 15–19 years (Ahmad, Boschi‐pinto, & Lopez, 2001).

### 2.7 Data analysis

Participants characteristics and adequacy of FV intake, leisure‐time PA, and engagement in sedentary behavior were presented as frequencies and proportions. Chi‐square (χ^2^) test statistic was used to determine the significance of association of selected factors with adequacy of FV intake, leisure‐time PA, and engagement in sedentary behavior. All statistical analyses were performed using the STATA 12.0 software program at *p* < 0.05 level of statistical significance.

## 3 RESULTS

### 3.1 Participants’ socio‐demographic factors

The study constituted of 621 respondents who completed the FV consumption tool (Table 1). Majority (54%) of participants were female, and most of the respondents (45.4%) were older adolescents (aged 15–19 years of age). There were more secondary (53%) than primary school participants. Majority (66%) of participants were from privately owned schools. The PA tool was completed by slightly more participants (652).

**Table 1:** Socio‐demographic characteristics of study participants

Item	Characteristics	*n* (%)
Gender	Male	287 (46.22)
	Female	334 (53.78)
Age category	8–9 years	68 (10.95)
	10–14 years	271 (43.64)
	15–19 years	283 (45.41)
Education level	Primary school	289 (46.54)
	Secondary school	332 (53.46)
School ownership	Public school	209 (33.66)
	Private school	412 (66.34)

Half of the participants (50%) reported inadequate fruit consumption and did not meet their daily recommendation. That is, they had consumed fruits less than two times per day in the past 30 days prior to the interview (Table 2). Majority of participants (25%) reported to consume fruits only once per day while 8% had not consumed fruit in the past 30 days prior to the interview.

**Table 2:** Frequency of fruit consumption among children and adolescents

Frequency of fruit consumption	*n* (%)
Did not eat fruit during the past 30 days	52 (8.37)
Less than one time per day	104 (16.75)
1 time per day	155 (24.96)
2 times per day	109 (17.55)
3 times per day	67 (10.79)
4 times per day	48 (7.73)
5 or more times per day	86 (13.85)

Adequacy of fruit consumption significantly varied with age category, school ownership, type of school, and participant's level of education (Table 3). A higher proportion of children (68%) than young adolescents (52%) and older adolescents (44%) met their daily recommendation for fruit consumption. More primary (59%) than secondary school (42%) and more public (57%) than private (46%) school participants met their daily recommendation for fruit consumption. Furthermore, more participants from schools that had both boarding and day sections (64%) than those who attended schools with only day section (48%) met their daily recommendation for fruit consumption.

**Table 3:** Association of selected factors with adequacy of fruit consumption among children and adolescents

Factors	Levels of fruit consumption		Chi‐square *p*‐value
	Adequate (%)	Inadequate (%)	
Age			
8–9 years	67.65	32.35	<0.01
10–14 years	52.03	47.97	
15–19 years	43.62	56.38	
Gender			
Male	49.13	50.87	0.72
Female	50.60	49.40	
Level of education			
Primary school	58.82	41.18	<0.001
Secondary school	42.17	57.83	
School ownership			
Public school	57.42	42.58	<0.01
Private school	46.12	53.88	
Type of school			
Day school	47.88	52.12	<0.01
Day and boarding school	64.10	35.90	
Total	49.92	50.08	

Overall, 24% of participants reported adequate vegetable consumption. That is, they met their daily recommendation for daily vegetable consumption of having consumed vegetables three or more times per day in the past 30 days prior to the interview (Table 4). Majority of participants (29%) reported to consume vegetables only once per day while 9% had not consumed vegetables in the past 30 days prior to the interview.

**Table 4:** Frequency of vegetable consumption among children and adolescents

Frequency of vegetable consumption	*n* (%)
Did not eat vegetable during the past 30 days	55 (8.86)
Less than one time per day	127 (20.45)
1 time per day	182 (29.31)
2 times per day	105 (16.91)
3 times per day	51 (8.21)
4 times per day	28 (4.51)
5 or more times per day	73 (11.76)

Adequacy of vegetable consumption varied statistically significantly with age category, school ownership, type of school, and participant's level of education (Table 5). While more participants reported adequate fruit consumption, the trends of adequate FV consumption were similar. A higher proportion of children (46%) than young adolescents (26%) and older adolescents (18%) met their daily recommendation for vegetable consumption. More primary (33%) than secondary school (17%) and more public (32%) than private (20%) school attending participants met their daily recommendation for vegetable consumption. Furthermore, more participants from schools that had both boarding and day sections (44%) than those who attended schools with only day section (22%) met their daily recommendation for vegetable consumption.

**Table 5:** Association of selected factors with adequacy of vegetable consumption among children and adolescents

Factors	Levels of vegetable consumption		Chi‐square *p*‐value
	Adequate (%)	Inadequate (%)	
Age			
8–9 years	45.59	54.41	<0.001
10–14 years	26.20	78.80	
15–19 years	17.73	82.27	
Gender			
Male	25.78	74.22	0.45
Female	23.19	76.81	
Level of education			
Primary school	32.53	67.47	<0.001
Secondary school	17.47	82.53	
School ownership			
Public school	32.06	67.94	<0.01
Private school	20.63	79.37	
Type of school			
Day school	21.73	78.27	<0.001
Day and boarding school	43.59	56.41
Total	24.48	75.52

Among participants aged 8–17 years, only 14% met their recommendation of 60 min of moderate‐ to vigorous‐intensity leisure‐time PA every day of the week during a typical week (Table 6). Majority of participants (21%) less than 18 years reported being active for a total of 60 min on only one day. A significant proportion of participants (11%) less than 18 years did not accumulate a total of 60 min of leisure‐time PA per day in a typical week. Among those above 17 years, 41% met their recommended weekly physical activity of 150 min of moderate‐ to vigorous‐intensity activity.

**Table 6:** Weekly leisure‐time physical activity levels in a usual week among children and adolescents

Number of days of 60 min of physical activity (PA) per day among children and adolescents aged 8–17 years	*n* (%)
0 day	64 (11.15)
1 day	121 (21.08)
2 days	91 (15.85)
3 days	74 (12.89)
4 days	67 (11.67)
5 days	33 (5.75)
6 days	41 (7.14)
7 days	83 (14.46)
Minutes of PA in a usual week among adolescents aged 17+ years	
<150 min	46 (58.97)
150 min or more	32 (41.03)

Adequacy of PA significantly varied with age category and school ownership (Table 7). A lesser proportion of 8‐ to 17‐year‐olds (14%) than 18 years and above olds (41%) met their weekly recommendation for moderate‐ to vigorous‐intensity leisure‐time physical activity. More private than public school attending participants met their weekly recommendation for moderate‐intensity physical activity.

**Table 7:** Factors associated with adequacy of leisure‐time physical activity among children and adolescents

Factors	Physical activity levels		Chi‐square *p*‐value
	Adequate (%)	Inadequate (%)	
Age			
8–17 years	14.46	85.54	<0.001
Above 17 years	41.03	58.97	
Gender			
Male	20.71	79.29	0.05
Female	14.91	85.09	
Level of education			
Primary school	20.42	79.58	0.10
Secondary school	15.47	84.53	
School ownership			
Public school	11.70	88.30	<0.01
Private school	21.71	78.29	
Type of school			
Day school	17.50	82.50	0.66
Day and boarding school	20.69	79.31	
Total	17.64	82.36	

Majority of participants (54%) did not spend excessive time in sedentary behaviors (Table 8). These did not spend more than 2 hr per day during a typical or usual day sitting and watching television, playing computer games, talking to friends, or doing other sitting activities, such as playing cards or board games or reading novels.

**Table 8:** Time children and adolescents usually spent on sedentary activities

Hours spent on sedentary activities per day	*n* (%)
<1 hr per day	133 (20.40)
1–2 hr per day	219 (33.59)
3–4 hr per day	145 (22.24)
5–6 hr per day	108 (16.56)
7–8 hr per day	24 (3.68)
More than 8 hr per day	17 (2.61)
Missing values	6 (0.92)

Acceptable level of sedentary behavior significantly varied with age category, gender, and level of education (Table 9). More of the older adolescents (85%) than young adolescents (80%) and children (52%) participated in excessive sedentary behaviors. More females (55%) than males (52%) and more students who attended secondary (62%) than primary (44%) schools engaged in excessive sedentary behaviors.

**Table 9:** Factors associated with participation in sedentary behavior

Factors	Level of sedentary behavior		Chi‐square *p*‐value
	Adequate (%)	Excessive (%)	
Age			
8–9 years	47.56	52.44	0.03
10–14 years	20.12	79.88	
15–19 years	14.52	85.48	
Gender			
Male	47.57	52.43	0.04
Female	44.74	55.26	
Level of education			
Primary school	56.06	43.94	<0.001
Secondary school	38.12	61.88	
School ownership			
Public school	42.26	57.74	0.11
Private school	48.58	51.42	
Type of school			
Day school	53.45	46.55	0.20
Day and boarding school	65.52	34.48
Total	53.99	46.01

When asked about what they felt after physical activity, majority of participants reported feeling refreshed and happy, some felt tired, and others did not do any PA while only 3% had other feelings (Table 10).

**Table 10:** Participants’ feeling after physical activity

Feeling after physical activity	*n* (%)
I don't do any physical activity	140 (21.47)
Tired	229 (35.12)
Refreshed and happy	261 (40.03)
Some other feeling	22 (3.37)

## 4 DISCUSSION

This study documents inadequate FV intake among the study population relative to international recommendations. Low intakes were observed both in fruits and vegetables and in all genders, in age groups, in primary and secondary, in public and private, and in day and day and boarding schools. Low‐ and middle‐income countries and high‐income countries have had the same trend of inadequate FV consumption levels among adolescents; for example, in seven African countries, 77.5% of adolescent students (Peltzer & Pengpid, 2012) and 78.6% of American high school students (Eaton et al., 2008) had eaten less than five servings of fruits and vegetables per day. This is similar to findings of other studies such as one conducted among Nigerian adolescents (Ilesanmi, Ilesanmi, & Ijarotimi, 2014). Up to a quarter of respondents had not taken fruits a month prior to the study and among those who had taken fruits, the intake was grossly inadequate (Ilesanmi et al., 2014). Findings are also similar to those obtained from school children aged 13 to 15 years from five Southeast Asian countries where the mean daily servings of fruits and/or vegetables per day was 3.2, far below the recommended five servings a day (Peltzer & Pengpid, 2012).

Inadequate fruit consumption could be attributed to participants’ perception of adequate consumption and about fruit market prices. A study carried out in Austria reported that the greatest barrier to higher FV intake was the perception that current individual consumption was already sufficient (Schätzer, Rust, & Elmadfa, 2010). Ghanaian students considered fruits expensive (Mintah et al., 2012). The inadequacy could also be attributed to home environment (Ong, Ullah, Magarey, Miller, & Leslie, 2017) nutrition transition happening in many sub‐Saharan African countries (Steyn & McHiza, 2014) including Uganda. Nutrition transition: the changes in dietary patterns and nutrient intakes when populations adopt modern lifestyles during economic and social development, urbanization, and acculturation (Vorster, Kruger, & Margetts, 2011). The process results in staples and side dishes being replaced by diets containing higher proportions of fats and meat, and less fruits, and vegetables (Steyn & McHiza, 2014). The change is associated with the documented increases in NCDs (Vorster et al., 2011). The reported inadequacy of FV consumption could result into an increased BMI and poor immunity among the study population due to deficiency of vitamins, minerals, fiber, and other dietary requirements, including photo‐chemicals with protective effects (Ramírez‐Silva et al., 2009). It could also result in, primarily or secondarily, more incidences of cardiovascular disease, type II diabetes mellitus, and several types of cancer (Ramírez‐Silva et al., 2009). FV consumption could be improved through ensuring a supportive social and physical environment, interesting children and adolescent in FV consumption as reported from a study among American students (Kimmons, Gillespie, Seymour, Serdula, & Blanck, 2009).

The current investigation also explored the prevalence of leisure‐time physical inactivity and sedentary behavior and their relationship to selected factors among school‐going children and adolescents in Kampala, Uganda. The inadequate levels of PA reported by this study are also common in other African populations. These were also reported in a study that assessed leisure‐time PA and sedentary behavior and substance use among in‐school adolescents in eight African countries (Peltzer, 2010). These results compare with other reports from mainly low‐ and middle‐income countries (Guthold, Cowan, Autenrieth, Kann, & Riley, 2010) (Peltzer, 2010). Since there is a negative association between PA and body mass (Peltzer & Pengpid, 2016), the high prevalence of physical inactivity is likely to result into increased body mass among school children and adolescents in Uganda. In order to improve cardio‐respiratory and muscular fitness, bone health, and cardiovascular and metabolic health biomarkers, children and youth should accumulate at least 60 min of moderate‐ to vigorous‐intensity physical activity (WHO, 2017b). Therefore, the inadequacy of physical activity could negatively affect children and adolescents’ health. The association between substance use and sedentary behavior has been reported by number studies (Iannotti, Janssen, Haug, Kololo, & Annaheim, 2010; Nelson, 2006; Peltzer, 2010). Association between loneliness and sedentary behavior and association between suicidal ideation and physical inactivity have been confirmed among girls (Peltzer & Pengpid, 2016). Generally, there is evidence of a protective association between physical activity and depression (Liu, Wu, & Yao, 2016). As a result of the high rates of physical inactivity and sedentary behavior reported among the in this study, there is a likelihood for loneliness, depression, substance use, and suicidal ideation in the study population and other similar populations.

On the other hand, a positive association between attending physical education classes and physical activity has also been found (Tassitano et al., 2010). Furthermore, the introduction of movable/recycled materials could have a significant, positive long‐term intervention effect on children's physical activity (Hyndman, Benson, Ullah, & Telford, 2014). Physical activity can possibly be increased by empowering and promoting physical education classes in schools and by introducing movable/recycled materials in school playgrounds. It was revealed that boys were more physically active than females. This higher PA may reflect a greater interest of boys for body fitness and building than females. Since majority of participants reported feeling good and happy after PA implying that interventions to promote PA could be embraced.

This study had some limitations. Firstly, it only included children and adolescents who were attending schools in urban Kampala district, which is not representative of all children and adolescents in Uganda. There may be differences in the occurrence of FV consumption, PA, and sedentary behavior between school‐going and non‐school‐going children and adolescents as well as rural and urban populations. As questionnaires were used for participants to self‐report their FV consumption, PA, and sedentary behavior, it is possible that some study participants biased their responses for example, over reporting FV consumption and PA. More valid measures for FV consumption and PA such food dairies and accelerometers could be used in future studies, to ensure more accurate data. Furthermore, this study was based on data collected in a cross‐sectional survey, and no causal conclusions can be drawn.

## 5 CONCLUSIONS

This study revealed that the majority of school children and adolescents still fall short of achieving the dietary recommendations for FV intake and PA and a significant proportion participate in excessive sedentary behavior. Age, level of education, gender, type of school, and school ownership were likely to determine FV intake, PA, and level of participation in sedentary behavior. Using the results of this research, the development and implementation of programs, strategies, and policies aimed at improving FV intake and PA among school children and adolescents in Uganda are recommended. This could be done through (a) information and communication strategies aimed at improving awareness of benefits of adequate FV intake and PA, (b) improving the availability and lowering the prices of FV for easier access by children and adolescents who may not afford higher prices, and (c) ensuring that physical education is part of both primary and secondary schools’ curricula. Multisectorial partnerships are also important and necessary to create supportive environments for the effective implementation of FV and PA promotion initiatives across different levels, including school, community, and national levels.

## CONFLICT OF INTEREST

The authors declare that they do not have any conflict of interest.

## ETHICAL REVIEW

Study procedures were approved by Makerere University School of Biomedical Sciences Higher Degrees, Research and Ethics Committee, and Uganda National Council for Science and Technology with approval numbers: SBS 291 and HS 1950, respectively.

## INFORMED CONSENT

The purpose and objectives of the study were carefully explained to each participant and their parents. Informed consent to the study was obtained from participants’ parent/guardian to affirm their willingness or not. The parents or guardians of participants provided consent to allow their children to take part in the study while participants signed assent accepting to participate in the study. The study conforms to the Declaration of Helsinki, US, and European Medicines Agency Guidelines for human subjects.
